# A Meta-Review of Indoor Positioning Systems

**DOI:** 10.3390/s19204507

**Published:** 2019-10-17

**Authors:** Germán Martín Mendoza-Silva, Joaquín Torres-Sospedra, Joaquín Huerta

**Affiliations:** Institute of New Imaging Technologies, Universitat Jaume I, Avda. Vicente Sos Baynat S/N, 12071 Castellón, Spain; jtorres@uji.es (J.T.-S.); huerta@uji.es (J.H.)

**Keywords:** indoor positioning, indoor navigation, smartphone-based positioning, meta-review, surveys, citations

## Abstract

An accurate and reliable Indoor Positioning System (IPS) applicable to most indoor scenarios has been sought for many years. The number of technologies, techniques, and approaches in general used in IPS proposals is remarkable. Such diversity, coupled with the lack of strict and verifiable evaluations, leads to difficulties for appreciating the true value of most proposals. This paper provides a meta-review that performed a comprehensive compilation of 62 survey papers in the area of indoor positioning. The paper provides the reader with an introduction to IPS and the different technologies, techniques, and some methods commonly employed. The introduction is supported by consensus found in the selected surveys and referenced using them. Thus, the meta-review allows the reader to inspect the IPS current state at a glance and serve as a guide for the reader to easily find further details on each technology used in IPS. The analyses of the meta-review contributed with insights on the abundance and academic significance of published IPS proposals using the criterion of the number of citations. Moreover, 75 works are identified as relevant works in the research topic from a selection of about 4000 works cited in the analyzed surveys.

## 1. Introduction

It is difficult to imagine our lives without positioning systems giving us support. GPS or similar GNNS constellations have shaped our modern life. From direction indications in a tourism trip to personalized advertisements, many of us enjoy the benefits of the development of Location-Based Services (LBS). Apart from GNNS services, LBS has been boosted by smartphones popularity [1,2]. An increasing number of the world’s population owns a smartphone and has Internet connectivity. People want their services provided in their smartphones and have their smartphones next to them at almost all times, they create excellent possibilities to deliver LBS. Position is also important for research in certain fields, such as medicine [3]. The global market knows that, and it is why LBS and their supporting technologies draw a lot of interest and investment [4,5]. In developed countries, people spend most of their time indoors. As the whole world develops, this tendency will expand to other countries. However, the position estimation indoors still lacks a generally applicable, low-cost solution that allows LBS tailored for indoor scenarios.

The position estimation of subjects, mainly mobile application users, in indoor environments is the topic of lots of works in academia, ranging from bachelor projects to doctoral dissertations to well-funded cross-national projects. The number of publications on the topic, of varying quality and in a variety of media, is so large that it is difficult to fully survey them all. It is also difficult to find all the reasons behind this thirst for publishing about indoor positioning. Some likely answers may include a general urge for publication in academia; a variety of new publication media; the ease of implementing a simple and cheap positioning system (thus encouraging its further research and publication); the elusiveness of a system that is accurate, cheap and applicable to many indoor environments at the same time; and the promise from industry of rapid adoption and billionaire investments for such golden system.

This work contributes with a meta-review (a systematic review of systematic reviews) of IPS research, deepening on Bluetooth Low Energy (BLE)-based and WiFi-based fingerprinting for smartphones. The meta-review addressed 61 selected surveys of IPS topics. Unlike regular reviews, our meta-review addresses topics of IPS from the point of view of surveys and not specific works. Every definition or explanation is the result of a consensus found on, or inferred from, several reviews. The paper provides three description sections, devoted to the introduction to IPS, a review of technologies used in IPS, and brief descriptions of methods and challenges for WiFi-based and BLE-based IPS for smartphones. The explanations in the description sections were supported, whenever possible, using references to the selected surveys works. Thus, the compiled meta-review allows the reader to view IPS current state at a glance and have direct links to deepens on the technology of her/his choosing. The meta-review detected that, currently, no survey focuses on BLE-based indoor positioning or radio map enrichment methods. Thus, this paper included brief reviews of proposals that belong to those two topics. The description sections are followed by a discussion section, in which the abundance and academic significance of published IPS works are analyzed. The analyses were performed based on the number of citations of each work, first attending to the references of the selected surveys and later using the Google Scholar citation counts for some selected works. The results showed that most of the works referenced in the surveys lack a significant impact on the IPS literature. Therefore, the three main contributions of this work are:

- A curated compilation of IPS surveys published in the last 5 years.
- Brief reviews of IPS solutions that addressed BLE-based indoor positioning and radio map enrichment methods.
- An analysis of IPS current state based on citations to works found in the selected surveys.

The paper proceeds with a short introduction to indoor positioning systems (Section 2), including the definition of accuracy and other IPS evaluation metrics. Later, Section 3 describes the main technologies used for indoor positioning, including the description of techniques and some methods applied to each of them. Section 4 then addresses smartphone-based indoor positioning using WiFi and BLE fingerprinting, including its associated challenges. Later, Section 5 performs the comprehensive analysis over 62 well-known surveys that draws insights on the works’ academic impact. Finally, Section 6 presents some concluding remarks.

## 2. Positioning in Indoor Scenarios

Positioning systems can be global or local. Global positioning systems can provide position estimations world-wide. The global positioning systems currently available are collectively called the Global Navigation Satellite System (GNSS). The GNSS-based positioning has had large success as a result of its availability, coverage, and the existence of receivers that are both cheap and compact-sized. However, it is not adequate for all scenarios and applications because of accuracy requirements and the degradation of satellite signals. Local positioning systems are the ones applied for those specific scenarios and applications where GNNS positioning is not appropriate. The locality or coverage of those positioning systems varies significantly. They range from systems based on networks of pseudolites that can cover very large areas to light-based systems that are typically applied to rooms. This paper focuses on local positioning systems indented to provide position estimation inside buildings, which are known as Indoor Positioning Systems.

The materials and structures of modern buildings may influence notably on the signals from GNNS. At indoors, those signals reach receivers with a level of degradation that makes the civilian-graded accuracy of GNSS insufficient for many indoor applications. Furthermore, indoor environments are normally crowded with fixed and moving obstacles, including people. The obstacles interact in undesirable ways with the signals, causing reflections and absorption. Given that people are increasingly spending most of their time indoors [6], the large efforts devoted along the past 15 years to find new solutions to IPS seems reasonable.

**Figure 1:**
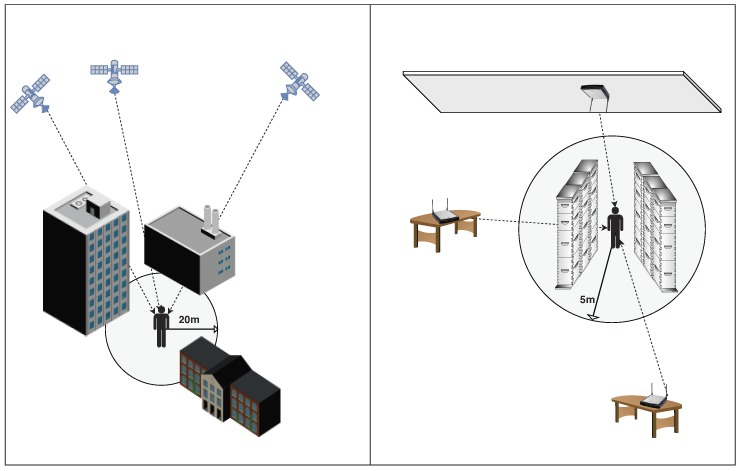
The magnitude of a positioning error matters differently to a person depending on the context.

Even if GNSS signals were not so significantly degraded by buildings, local positioning systems will still be needed for indoor LBS applications. Some applications require positioning accuracies far beyond those that GNNS can provide. For example, a robot performing precise operations and whose next move could not be determined in an offline planning step will probably require a millimeter level accuracy. Furthermore, the accuracy requirements for pedestrian applications may be related to the size of the target environment. The comfort thresholds of people regarding the positioning accuracy can be related to the free space they have around, as depicted in Figure 1. The typical accuracy for a smartphone’s GNSS receptor under open sky conditions is 4.9 m [7], which is fine for someone at a park or wide street. However, that distance may be misleading in indoor environments. For example, a mean accuracy of 5 m would be insufficient to tell apart consecutive lanes among bookshelves in a library, which sometimes are less than 1 m apart from each other. Therefore, an IPS applicable to most indoors environments and applications would preferably have mean accuracies around 1 m to 2 m.

Accuracy is the degree of conformance, or the closeness, between an estimated or measured position and the true position of a subject or object at a given time [8]. The above accuracy definition is wide. However, given the significant number of different existing (indoor) positioning systems, it is difficult to have a narrower definition or only one metric [9,10]. Even though accuracy is of utmost importance, it is not the only criterion taken into account for assessing an IPS. Coverage, complexity, robustness, scalability, cost, privacy and power consumption are metrics typically used for IPS evaluation and comparison [11,12,13,14,15]:

- Coverage refers to the range of the signals from the technology that supports an IPS [11]. A high coverage can translate into the IPS’ applicability to large areas using a low number of emitters [15].
- Complexity refers to the efforts required for the construction, deployment or configuration of the hardware and software of the IPS [12,13,14].
- Robustness is the system’s resilience to conditions beyond those that are considered nominal [12].
- Scalability relates to the system’s ability to provide positioning for a large number of users in large spaces [12,15].
- Cost refers to any kind of cost related to the positioning devices or the required infrastructure [11,12].
- Privacy is related to system restrictions that avoid the collection of information that may be used to identify or track the users [11,13,14].
- Finally, the lower power requirements, the better. In user devices, a low power requirement translates into a low battery drain. For the IPS infrastructure, a low power consumption may translate into that only small efforts are required for the maintenance of, e.g., battery-powered devices [11,15].

## 3. An Overview of IPS Solutions

The high demand for IPS has driven an increasing number of research works along the past 15 years or more. The number of IPS proposals found in this period is very large, which is reflected in the number of references of recent IPS-related survey works. Some IPS-related reviews have over 200 references [16,17,18,19,20,21] or even over 300 references [22]. The insights presented in the rest of this paper are mainly based on 62 IPS-related survey works [6,11,12,13,14,15,16,17,18,19,20,21,22,23,24,25,26,27,28,29,30,31,32,33,34,35,36,37,38,39,40,41,42,43,44,45,46,47,48,49,50,51,52,53,54,55,56,57,58,59,60,61,62,63,64,65,66,67,68,69,70,71,72] published between 2015 and 2019. Of them, 47 were published in periodic publication journals. Surveys that only briefly mentioned indoor positioning techniques or whose content was not available online, such as Aparicio et al. [73], were not considered. Table 1 shows the number of the selected surveys that were published each year.

The notable number of surveys has driven them into narrow focuses. However, some of them have addressed a broad spectrum of solutions applicable to indoor positioning [11,12,13,14,15,45,46,47,48,57]. It is common that these “general” surveys difference from previous surveys not only by providing updates of new IPS solutions but by proposing new taxonomies or by discussing more than others about specific aspects such as specific applications or challenges. Xiao et al. [45] provides a valuable division between device-free and device-based IPS solution across several technologies. [47] states that hybrid systems are the ones most suited to mass-market applications, and thus focus on reviewing some solutions that combine several techniques. Yassin et al. [14] devotes a section to limits of positioning, though it is brief and has a small number of references to other works. It also features a section devoted to cooperative positioning and data fusion, which provides interesting examples but lack of a clear definition that separates the two concepts. Sakpere et al. [13] is relatively recent, deals with almost all technologies, makes no restriction on specific applications, has a large number of references and discusses the challenges and drawbacks of each technology. Brena et al. [46] is comprehensive, and tells apart “passive” from “active” solutions, considering passive those in which only the infrastructure generates the signal used for positioning. Basiri et al. [11] provides a review of the IPS research status supported by a literature review and a survey whose responders were mainly LBS ordinary users, LBS researchers, LBS market analysts, and LBS application developers. Such a survey was a necessity, given that the usages and goals of IPS are clearly beyond the published academic works. Zafari et al. [15] is, to the best of our knowledge, the most recent IPS survey (2019). This survey stands out for its Internet of Things (IoT) flavor that links IoT and indoor positioning. Also, it devotes a section to IPS applications and provides a summary of the main IPS challenges and their suggested solutions.

Other surveys restricted the reviewed solutions by technology. For example, Maghdid et al. [41] and Davidson and Piche [48] described solutions that were applicable to smartphones. In particular, Davidson and Piche [48] is relatively recent which is remarkable because of conciseness that properly weights explanations and references, while addressing every major aspect of smartphone-based IPS. If the goal of the reader is positioning for smartphone applications, Davidson and Piche [48] is recommended as a first reading.

There is not a clear consensus among this “general”, or other, surveys on a strict taxonomy for IPS. The most commonly found classification attends to the underlying technology. The technology then determines which physical quantities the system can measure. The physical quantities are also used for IPS classification and are commonly addressed as the applied techniques. On top of technologies and techniques, there is a myriad of methods that, from the measured quantities, create position estimations. The methods also tend to be classified into range-based or range-free methods, depending on whether they estimate distances or angles to known landmarks like, e.g., the signal emitters.

The following list briefly introduces the most common techniques employed with technologies used in IPS. They are addressed in almost all the selected surveys and they are often mentioned in the rest of this section. Some general surveys [12,22,41] include explanation and usages of other techniques such as Received Signal Phase (RSF), Roundtrip Time of Flight (RTF) or Channel State Information (CSI).

- Time of Arrival (TOA). It measures the time of arrival of the signal from an emitter, as recorded by the receiver. It is used for estimating the distance to each emitter, as the propagation speed of the signal (sound, radio frequencies) is known for the transmission medium (air).
- Time Difference of Arrival (TDOA). It is similar to TOA. It measures the differences in the time of arrival of signals from different emitters. It is used for estimating differences in distances to each emitter.
- Angle of Arrival (AOA). It refers to the angle at which the signal reaches the sensor. Angles are then used to obtain a position fix.
- Received Signal Strength (RSS). It is the intensity at which the signal from an emitter is measured. The signal strength decreases as the distance to the emitter increases, although their relation may be affected by attenuation and interference.

**Figure 2:**
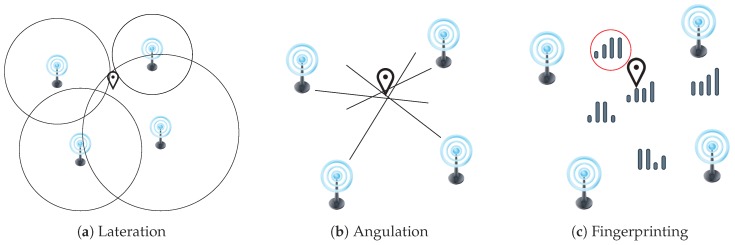
Most common methods used in IPS.

The technique employed for a solution determines how the position is estimated. TOA, TDOA, and RSS are used for estimating distances to signal emitters. The estimated distances to a set of emitters are then used in what is called lateration to find the position estimate that best fit the set of distances (see Figure 2a). Lateration is called trilateration if three distances are used, while it is called multilateration if more than three are used. The angles obtained in AoA are used to compute a likely fix on the target position, as shown in Figure 2b, in what is known as angulation. Both lateration and angulation are commonly classified as range-based—or ranging—methods, and they require the previous knowledge of the positions of the emitters.

The RSS technique is also employed for a range-free method, very popular in IPS, called fingerprinting or sometimes scene analysis. The fingerprinting encompasses two stages. In the first stage, also known as offline stage, the signal quantity of each detected emitter at a given time and position (a fingerprint) is measured at several places the target scenario and stored to create a characterization of the signals in that scenario as comprehensive as possible. The collected database is called the training database. If the measured signals are radio frequencies (RF), the database is also called radio map. The collection process of the database is called site survey, war-driving, radio map creation or training fingerprints collection. In the second stage, also known as online stage, the position corresponding to new measured signal quantities is estimated using the positions associated with the stored fingerprints that are the most similar when compared to the new measurements (see Figure 2c).

Apart from the previous methods, another two relevant ones are not based on measuring signals from an emitter and are also considered range-free methods: hop count and vision analysis. A hop occurs when a packet passes from a network segment to the next one. The number of hops from known nodes is then used for (coarse) distance estimations [18] or to infer positions through a graph embedding problem [20]. Vision analysis refers to the application of computer vision approaches to images gathered using some imaging technique like, e.g., cameras. The analyses detect relevant features in the scene that allow the estimation of the positions of entities in the scene or the position of the recording imaging device [74].

Another relevant high-level classification is device-based or device-free positioning [45]. In the former, there is a positioning device that acts as an active agent of the positioning process by measuring or emitting a signal. The positioning device is normally a portable device such as a smartphone or a tag. Device-based solutions are the most commonly addressed in surveys and IPS literature in general. Device-free positioning is found mainly in the form of presence detection or radar-like systems. The importance of the device-based vs device-free division is supported by the fact that one of the driven forces for the growth of LBS is the widespread usage of smartphones. Some surveys explicitly focus on IPS solutions applicable to smartphones [41,48] and others on device-free positioning [53,55]. The distinction on whether an IPS is applicable to smartphones or not is necessary given that even modern smartphones have a limited number of sensory capabilities. Another classification is whether the solution requires the deployment of dedicated infrastructure or not, being the former called an infrastructure-based solution and the later an infrastructure-free solution [6].

The underlying technology does matter, and its particularities should be taken into account when creating an IPS. This section proceeds by providing a review of the technologies most commonly applied for IPS solutions. In each case, relevant references to the applied techniques and the applicability to range-based vs range-free or device-based vs device-free classification will be mentioned.

### 3.1. Light

Visible Light Communication (VLC) IPS appear as device-based solutions, while infrared IPS may appear both as device-based or device-free (passive) solutions. The VLC-based IPS rely on the idea that LED lighting is increasingly popular, and LEDs can switch the intensity level in a way that is fast and imperceptible to the human eye. The intensity level switches are then used to encode information. Settings that have several LEDs emitters use a multiplexing protocol, either by frequency or time. The receiver is commonly equipped with a photodiode array that captures signal properties such as RSS, TDOA, and AOA, or with a camera that takes images of the transmitters. For the former, positioning approaches such as lateration, angulation or fingerprinting can be applied. For the later, the coordinates that the LEDs have in the image are translated into coordinates of the environment, usually using several images and additional support hardware such as accelerometers. The mean accuracies reported by VLC-based IPS are measured in centimeters [37,64,65]. However, as stated by Do and Yoo [37], the reported results should be taken with caution as the test scenarios and conditions vary significantly among these solutions. Also, challenges such as emitters time synchronization [24] and robustness to sunlight [37] remain.

Infrared signals have been also used for light-based IPS, either for device-based or device-free solutions. A notable example of the former is the pioneering work of the Active Badge Location System [75], where a tag emitted a code carried by an infrared signal that was captured by a network of sensors. Examples of the latter are Passive Infrared (PIR) solutions, which could use infrared cameras for imaging or thermopile arrays that provide AoA measurements [21,45,46,55]. According to the performed searches for this work, there is no infrared-based IPS survey and the number of such system proposals is low when compared to other IPS technologies, which may be motivated by the LOS requirement of infrared-based solutions.

Most of the light-based proposals agree that one of their motivations is the possibility of reusing existing LED lighting infrastructure. In particular, the consulted surveys expected a growth of VLC systems that would lead them to be used for positioning in a way similar to that of WiFi [24,37,51,64]. Some years ago, the capabilities of the smartphones were a concern for light-based IPS [24], which led to even performing some computations through external services [37]. Those capabilities are higher now, enabling camera-based designs and shifting the concerns to challenges such as power consumption of the camera, daytime lighting conditions, and accuracy improvement [64]. However, camera-based designs do not achieve the accuracies of photodiode-based designs and require LOS situations. Furthermore, VLC systems are not as ubiquitous as WiFi. Thus, light-based IPS are sometimes acknowledged to be in early stages of development [65]. The surveys of Zhuang et al. [64] and Afzalan and Jazizadeh [65] are comprehensive and recent enough to grasp general but solid knowledge of light-based IPS.

### 3.2. Computer Vision

Apart from their support in VLC-based or infrared-based positioning, vision techniques are also used in camera-based IPS without any supporting light framework apart from those common to any modern building. Even though Simultaneous Localization and Mapping (SLAM) is mainly based on cameras for sensory input presently, one should not enclose SLAM into vision-based methods. SLAM is possible using, for example, laser scanners, sonars or odometric data provided by wheel encoders [16].

For device-based solutions, one of the most straightforward solutions is based on markers like, e.g., printed QR codes. Marker-based IPS may even provide continuous estimations if the markers are processed in a stream of images (video) of the scene and perspective is used for refinement [13]. Visual odometry is commonly applied to the input from one or several cameras. The cameras can be monocular, stereo or omnidirectional. The cameras motion and subject speed are usually determined by applying methods such as feature tracking or optical flow [16,74]. Visual odometry surpasses other odometry technologies regarding cost and accuracy [74] and is supported by modern computer vision techniques and the computation power found in robots and mobile devices, such as in the applications of Google’s ARcore [76].

For device-free solutions, it is common that several cameras are installed in the target environment. The collection of images is then used to identify the targets in conjunction with environment details recorded for the target scenario [12].

Position determination using computer vision techniques is likely to be increasingly used in the near future. Apart from visual odometry and vision-based SLAM, other applications such as self-driving cars and immersive applications such as those of virtual and augmented reality in video games have boosted and will further boost the usage of computer vision to determine subject positions. Those applications have not only driven the development of software techniques but also specialized hardware such as depth cameras [16]. In addition, the drift provided by visual odometry is smaller than the drift of wheel encoders and low-precision Inertial Navigation Systems (INS) [74]. Despite being devoted to SLAM, the survey of [16] provides a necessary context to explore the application of computer vision techniques to positioning, from the perspective of 2016. Although [16] is a recommended reading, new survey works on this topic are required. Those works could include the examination of device-free solutions.

### 3.3. Sound

The selected surveys that focus on acoustic signals make a distinction between systems operating on ultrasound—frequencies beyond 20 KHz—and those using audible frequencies [13,45,46,61]. They reference as pioneering works the “Active Bat” [77], “Cricket” [78] and “Dolphin” [79] systems for ultrasound, and the “BeepBeep” system [80] for audible frequencies. The ultrasound-based IPS generally use TOA or TDOA to perform lateration, although RSS has also been used [21,46], even for fingerprinting [45]. To improve the accuracy, the ultrasound pulses are complemented with RF pulses [78] or BLE advertisements [81].

Even though the acoustic solutions have reported accuracies of a few centimeters [14], they present notable changes as sound speed is affected by temperature and humidity. Furthermore, they suffer from interference of bouncing pulses and, in the case of ultrasonic systems, they require specialized hardware that is expensive to deploy [15,61]. Most of the acoustic IPS presented in the surveys refer mainly to device-based IPS. There are examples of device-free IPS in the form of audible sound source positioning, and as ultrasonic radars [12], although they face strong challenges due to noises, echoes, and multiple sound sources [53].

Early proposals of acoustic-based IPS were published in the late 1990s and early 2000s [77,78,79]. However, they have not become largely adopted by IPS solutions, despite their high accuracy. They require deployments which are relatively cheap for just one room but whose affordability may become a concern for large environments. Also, the acoustic sensing capabilities of modern smartphones are not enough for the proposed acoustic-based IPS. Ureña et al. [61] provides a comprehensive and recent (2018) survey on acoustic local positioning systems, with a moderate number of references that should suffice as an introduction and update to works in this topic.

### 3.4. Magnetic Fields

Pasku et al. [54] classifies the magnetic-based solutions into those that use the natural Earth magnetic field, those that use DC (static) artificial magnetic fields, and those that use AC (time-varying) artificial magnetic fields. Other general surveys, such as Zafari et al. [15], do not make such distinction, while others such as Brena et al. [46] do make the distinction but concentrates on those that only use the Earth magnetic field, as a result of considering that it is the approach followed by most modern solutions. Indeed, the selection made by Brena et al. [46] is supported by the facts that such systems do not incur in deployment costs, they are readily applicable to smartphones [48], and their reported accuracies are in the range of a few meters.

Magnetic IPS use strength variations in the measured magnetic field to infer a position estimate. In the case of the ambient (Earth) magnetic field, such variations are usually caused by steel structures in the target indoor scenario, requiring the creation of a database with the recorded variations of the magnetic field strength [6]. The created database is later used by a fingerprinting method to compute position estimations. One of the most known works using this approach is Magicol [82], which is referred by several surveys [12,48,54].

IPS based on the Earth magnetic field require a collection effort and typically have lower accuracies than those based on artificially generated magnetic fields. However, they have a low cost, a low system complexity, and a large operating range [48]. Those based on artificial fields require coil-based systems that are power hungry and operate at short ranges, but they are able to produce accuracies in the range of several centimeters [54]. All magnetic-based IPS are device-based IPS solutions, to the best of this work’s knowledge.

IPS based on artificially generated magnetic fields still require the miniaturization transmitters and receivers and the reduction of the power consumption Pasku et al. [54]. Pasku et al. [54] reported commercial systems from 2013 to 2016, mainly devoted to artificially generated magnetic fields. Pasku et al. [54], despite providing a comprehensive general introduction and update to magnetic-based IPS, devotes more content to the artificial magnetic fields IPS than to the naturally occurring ones. Naturally occurring magnetic fields are mainly used in IPS intended for smartphones, and thus the survey presented in Davidson and Piche [48] is recommended as a brief complementary reading. For a survey of the application of Earth magnetic field to smartphones-based IPS, He and Shin [50] is recommended. That survey explains how the measurements are obtained, how the database is created and how the positioning is performed, presenting for each aspect relevant and recent—for its time—works. Also, the survey is comprehensive and presents measurement ranges from several devices.

Given that IPS that use Earth magnetic fields are common in IPS literature, mainly combined with other technologies such as WiFi and Pedestrian Dead Reckoning (PDR), and He and Shin [50] reviews works mainly from before 2017, a new magnetic-based survey is suggested. The survey should provide an update on academic and commercials proposals based solely on magnetic fields and an update on proposals that combine Earth magnetic fields with other technologies, explaining how the combination is performed.

### 3.5. Dead Reckoning

Dead Reckoning (DR) is applied to device-based IPS. When specifically targeted for pedestrian, it is called Pedestrian Dead Reckoning (PDR). Dead Reckoning refers to the estimation of the current position of a target based on a previously known position (a fix) of it and measurements of quantities that are used to describe its movement, e.g., heading and speed [13]. Such measurements are commonly obtained using accelerometers that sense translations, i.e., provide the acceleration magnitude at each of the three axes; gyroscopes that sense rotations, i.e., provide roll, pitch and yaw measurements; and magnetometers (compasses, which are not inertial sensors) that give orientation regarding the Earth magnetic poles, i.e., provide field strength measurement along three axes.

Modern smartphones include most of those sensors and have the computing capabilities to perform PDR. PDR works have also used units that assemble inertial sensors, which are called Inertial Measurement Units (IMUs). IMUs are mounted mainly in feet and legs, although it has been reported also for waist mounts [62,70]. The shoe-mounted setting has been the most popular, given that the mechanics involving the walking process and the foot allow re-calibrations at every step applying the Zero-velocity UPdaTes (ZUPT) method [70]. Filtering algorithms such as (Extended or Unscented) Kalman Filtering and Particle Filter (PF) that combine all information are at the core of DR [13,62].

The movement or trajectory is usually estimated using two approaches. The systems that use the first approach are called Inertial Navigation Systems (INSs), and they perform the integration of the sensor data. The systems that used the second approach are called Step and Heading Systems (SHSs), and they detect and quantify steps and their headings [12,13,62,83]. If possible, other inputs such as maps and constraints are applied using filters to improve the resulting accuracy [62]. PDR has a low cost, it does not require external references, and it has a high accuracy when new positions are not estimated far apart from the last fix. However, it suffers from accumulative errors or drifts [13]. That is why PDR is usually used coupled with other technologies that support IPS and provide periodical estimations that help in correcting drifts.

Dead reckoning is widely used in position estimation and navigation systems, not only for indoors. Unless a system can deliver very high positioning accuracies with high certainties for moving targets, it will certainly benefit from using DR. Similarly, PDR for smartphone-based IPS benefits from fixes provided by other supporting technologies. The smartphone’s IMU sensors have limited accuracy in comparison to other (bigger and more expensive) IMUs, which make the accumulated drift to become a problem [62]. However, miniaturized IMUs are improving, as seen in the now popular mHealth gadgets, which will make PDR less dependent on other technologies to solve drift problems [70]. Despite the survey of Diaz et al. [70] is very recent (2019), the works of Wu et al. [62] provides a recent (2018) and more comprehensive survey about PDR based solely on inertial sensors. To explore the combinations with other technologies for the case of smartphone-based IPS, Davidson and Piche [48] is suggested as reading. The work of Vezocnik and Juric [71] is recommended for an exhaustive and up-to-date survey on step length estimation models, a key part of many PDR solutions.

### 3.6. Ultra-Wideband (UWB)

UWB is highly acknowledged as an IPS technology. The three UWB surveys [30,33,52] agreed in using the USA Federal Communications Commission (FCC) definition of UWB, which states than it refers to RF signals whose bandwidth is greater than 20% of the center carrier frequency, or is greater than 500 MHz. That large bandwidth is related to a key characteristic that is acknowledged in many works: UWB works by emitting precisely timed very short pulses, of ≈200 ps (pulse width), with very low transmission power [84]. The low transmission power avoids interference to WiFi, BLE or similar. The very short pulse modulation renders UWB almost immune to multipath issues. Given that the inter-pulse period is large enough to unambiguously perform multipath resolution, NLOS paths are detected after the main pulse detection [52]. Also, UWB has penetration capabilities considerably larger than WiFi and BLE, being LOS situations less relevant for it [33]. Furthermore, the energy consumption is lower than other WLAN technologies such as Bluetooth or WiFi [52].

UWB device-based positioning requires the deployment of tags. UWB emitters have been reported either in fixed or mobile configurations, with tags having different sizes and shapes and being mounted or worn at different places on the positioning subject, e.g., mounted on the feet or the head [21,46]. With UWB, the positioning is performed using any of the RSS, ToA, AoA, or TDoA techniques, depending on the tags design and their resulting capabilities. Therefore, the reported methods used for positioning are fingerprinting, lateration or angulation [30,33,52]. Given the effort implied in the radio map creation for fingerprinting, it is the least used method for UWB. The reported accuracies are typically below the 50 cm [52], which makes UWB attractive for many applications, as long as they can afford the UWB tags, both in terms of cost and the application requirements. However, the short nominal range of UWB [52] and the cost of UWB equipment [33] makes the scalability a severe issue.

For device-free positioning, UWB has been used by applying the principle of radar [33], which is attractive given the UWB capability of penetrating through walls. In a room with UWB emitters and receivers, a subject creates reflections of the signals that, using the TOA and TDOA techniques, can be used to estimate the subject’s position [12,13].

One of the biggest hurdles for UWB is the lack of support for it in almost all smartphones, apart from some specific attempts [85]. However, a recent announcement of the incorporation of UWB chips inside new versions of Apple’s iPhone [86] may change the IPS landscape, at least for small scale scenarios. Many customers will choose deployment cost higher than WiFi or BLE to obtain significantly higher accuracies.

Regarding reading suggestions, Mazhar et al. [52] provides a short but helpful comparison of BLE, WiFi, ZigBee, and UWB and explains the advantageous multipath resolution in UWB. Ref. [52] is suggested as introductory reading to UWB-based IPS that can be deepened by reading Alarifi et al. [33]. Given that Alarifi et al. [33] and Mazhar et al. [52] were published in 2016 and 2017, a new survey on UWB-based IPS will have a notable value. However, its value would be remarkably high if include foreseen applications of the new UWB chips of Apple’s iPhone.

### 3.7. WiFi

WiFi, or Wi-Fi, is the IEEE standard 802.11 for WLAN [27]. WiFi-based positioning is sometimes addressed by the name of WLAN positioning [13,14,46], which is the result of WiFi being the default technology for setting up a WLAN. WiFi is mentioned as an IPS supporting technology in all selected surveys. Furthermore, to the best of this work’s knowledge, it is acknowledged in all published IPS proposals.

WiFi operates on the of 2.4 GHz and 5.0 GHz [27], with typical channel widths of 20 MHz, 40 MHz, and 80 MHz. The signals from bands of 2.4 GHz travel farther, while the those from bands of 5.0 GHz have wider channels and are more robust to fast fading [87]. Works that report the usage of CSI, ToF and AoA techniques are not uncommon [15]. However, the main applied technique is RSS, given that is the technique applicable to many modern smartphones [48]. Although they are very popular, IPS based on WiFi have many challenges that arise from the alterations that the RF signals suffer in indoor environments. Also, the WiFi-based proposals use the existing WiFi networks to achieve low-cost solutions, but those networks are commonly deployed for communication purposes and not for positioning [15]. Solutions for WiFi RSS positioning may apply lateration based on a propagation model if the AP positions are known, which is also known as model-based approach [19]. However, the most popular approach is fingerprinting [46] because of its consistently better accuracy results in comparison to lateration. Given their direct applicability to many smartphones, WiFi-based IPS are mainly device-based solutions.

There are also device-free solutions [88], which commonly perform anomaly or motion detection first and then determine the position of the entities using techniques such as RSS or CSI [45]. Fingerprinting, link-based schemes, and Radio Tomographic Imaging are among the employed methods [45,88].

Along the years, WiFi-based IPS surveys and solutions have acknowledged the known challenges and forecast improvement actions. However, is not commonly recognized that the widely acknowledged typical accuracies of WiFi-based IPS have not significantly improved in recent years. Forecast techniques such as CSI [17] have not finally had a large success. WiFi-based IPS, mainly those that use fingerprinting, have enjoyed popularity because they are cheap and easy to implement. However, its popularity may be affected by new changes to Android smartphones [89].

We recommend the reading of Khalajmehrabadi et al. [19], as it provides an easy to follow survey that addresses the most important works by the time of its publication. The survey from Khalajmehrabadi et al. [19] is as comprehensive and more recent than that of He and Chan [17]. Also, it provides a summary of the reported accuracy of several solutions along with the testbed conditions. Makki et al. [27] and Konings et al. [88] provide surveys of the very specific topics of WiFi device-free and WiFi time-based solutions, respectively. The survey from Konings et al. [88] is recent (2019) and provides experimental result obtained by the authors. The publication year of the main surveys that addressed WiFi fingerprinting is 2016 for He and Chan [17] and 2017 for Khalajmehrabadi et al. [19]. Thus, a new survey on WiFi-based IPS is suggested. The new survey should provide a general inclusive overview of WiFi-based solutions, which is missing from the consulted surveys. However, it should devote most of its content to WiFi fingerprinting, given the popularity of this topic.

### 3.8. Bluetooth Low Energy (BLE)

To the best of this work’s knowledge, no survey focuses on BLE-based IPS currently, although surveys covering wider BLE topics do exist [90]. BLE follows Bluetooth classic in the usage of frequency hopping to communicate. The BLE emitters are commonly called BLE beacons. They are small, advertisement-emitting devices available in many configurations that are attractive for IPS given their cost, privacy, and a low footprint on the smartphone’s battery and network traffic [48,87]. BLE advertises at three channels of 2 MHz of width in the 2.4 GHz band [87]. The small channel width translates into a larger fast-fading effect than that seen for WiFi, even for the 2.4 GHz band [87]. Thus, solutions to reduce the fast-fading effects on BLE-based IPS are very relevant [91]. Nevertheless, BLE is sometimes seen as the most suitable positioning technology for indoor navigation and tracking currently [11]. Its suitability is supported by the relatively low cost of BLE emitters, their very low power consumption that let them run on batteries for months, and a generalized capability of modern smartphones to read their advertisements [46].

BLE shares many similarities with WiFi at the 2.4 GHz band [48], and thus it has been used for positioning by applying the RSS, AoA, and ToF techniques, being RSS the most often applied technique [15]. To the best of this work’s knowledge, BLE has been used only for device-based IPS. BLE has a low detection range, typically under 20 m [11]. Such a short detection range reduces its applicability to device-free solutions. The accuracies achievable using BLE is typically higher than those of WiFi, which is related to the usually higher density of deployed emitters [48]. Even though BLE beacons are generally cheap, the scalability to large scenarios may be an issue if dense deployments are required [13]. Given the lack of BLE IPS surveys, we provide here a brief review that addresses topics that we considered to be the most relevant for BLE-based positioning.

Attempts to use Bluetooth for indoor positioning date back to the early 2000s, using proximity [92,93] and lateration [94]. However, scanning times were too large [95], thus rendering Bluetooth not suitable fine-grained low latency positioning [96]. After the development of BLE (2012), the iBeacon specification by Apple, and the increase in the supply of cheap BLE beacons, Bluetooth (BLE) notably increased its popularity for positioning.

One of the most important early works on BLE for positioning is Faragher and Harle [87]. That work showed a BLE comparison between WiFi and BLE. Also, it showed how BLE measurements are affected by an uneven channel gain (which creates different measurements for different channels). Thus, it showed one of the most important challenges of BLE positioning, a fast-fading effect that is more significant than the one seen for WiFi. Also, Faragher and Harle [87] gave guides on the usage of a moving window for averaging measurements and improve signals measurement errors, attending to criteria such as possible walking movement, fast fades, and client advertisements receive rate. Palumbo et al. [97] proposed a method that dealt with BLE fast fades by using a map that simulated a trail diffusion. Kriz et al. [98] showed the relation between higher advertisement frequency and denser beacon deployments to better the positioning accuracy.

Unlike WiFi, with BLE it is possible to decide the deployment of the emitters considering only the positioning purpose. However, it creates the challenge of finding the most suitable combination of beacons and the broadcasting settings for a given environment. Oftentimes, the environment creates hard restrictions on where it is possible to deploy the beacons, and sometimes a uniform deployment is just assumed. Faragher and Harle [87] acknowledged that an exhaustive search of the parameter space was infeasible, and the authors chose a convenient deployment for the environment that set an upper bound on the positioning error after trying several parameters that included moving windows size, windows aggregation method (mean, median and maximum), beacon advertising frequency, and transmission power (beacons range). Budina et al. [99] proposed a method of iBeacon optimal distribution for indoor localization, where a predefined number of beacons are situated over the area, so their coverage is optimized. The procedure they proposed divides the target area into cells for their independent evaluation and take into account the building layout. They stated that optimization is in line with detecting enough devices with enough signal intensity. Castillo-Cara et al. [100] studied the beacon setup parameters, namely transmission power, density, and topology. They recommended splitting the target area into large sectors, keeping gaps separating areas between them to improve the classification. Also, they suggested to use low or medium transmission powers and to take into account the materials composing walls and avoiding positions near windows. Also, they recommended placing beacons at the corners and one in the center of the area, with a beacon at least every 6 m. In their experiments, they tested a grid deployment. Newer proposals include the work of Rezazadeh et al. [101], which analyzed in an environment the vertical and horizontal positions of beacons to increase the measured intensity of each beacon and thus increase the chances of seeing at least three of them at any position.

With WiFi, the default approach to use is fingerprinting. However, a higher density of emitters and their shorter detection ranges allow other strategies for BLE depending on the deployment and environment. Aman et al. [102] used the center of the bounding box of the detected beacon positions as the position estimate. Bouchard et al. [103] showed that higher values of mean RSS, received advertisement count ratios, and variance were correlated with shorter distances to a beacon. Thus, the authors chose the position of the beacon that had the highest value of the weighted combination of the three metrics as the position estimate. Muñoz-Organero et al. [104] tested the centroid of detected emitters, either giving or not predefined weights to each emitter. More recently, Mendoza-Silva et al. [105] tested the centroid of the positions of the detected beacons in two environments, weighing each position according to the RSS of the beacon. The accuracy obtained using the weighted centroid was higher than using fingerprinting. [97] applied a technique referred by the authors as stigmergy, which iteratively updates a map that simulates a trail diffusion created by a sequence of position estimates. Only a few works have used lateration in BLE-based IPS. [106] used it with specialized equipment (i.e., not smartphones or off-the-shelf BLE emitters) considering only LOS conditions with senders and receivers at the same altitude. Huang et al. [107] and Huang et al. [108] also used lateration, but improved the estimations using either PDR and Kalman filtering or a heuristic for improving the distance estimation through channel separation.

Fingerprinting has been an important approach for BLE positioning since its beginnings. As shown in Mendoza-Silva et al. [105], fingerprinting is a better option than proximity or lateration when the number of BLE beacons is small. Zhang et al. [109] used fingerprinting using two configurations of neural networks, Support Vector Machines (SVM) and k-Nearest Neighbors (kNN) with or without map enrichment with regression. SVN provided the best accuracy results. Zhao et al. [110] and Lohan et al. [111] presented comparisons between the usage of WiFi and BLE for positioning. In the case of Lohan et al. [111], the experiments considered propagation models, fingerprinting, and the weighted centroid method, with the latter providing the best accuracy results. Faragher and Harle [87] used Gaussian Process Regression for radio map enrichment. The entire area of interest was divided into grid cells of side 1 m. Map cells with moderate or high variance were ignored completely for the operational stage. The authors used a Gaussian Kernel posterior probability on each cell for the current fingerprint, and estimated the position using the maximum a-posteriori probability. Kriz et al. [98] tested the combination of WiFi and BLE under one distance in the signal space for kNN fingerprinting, giving the same weight to both measurements. Wang et al. [112] used kNN once the radio map was transformed using the Isomap-based manifold data dimensionality reduction technique. Castillo-Cara et al. [100] tested weighted kNN and SVM, and provided recommendation of their parameters when using low and high transmission powers. Zuo et al. [113] combined the fingerprinting and lateration approaches, and further improved the estimates by applying PDR.

Table 2 presents a summary of some of the BLE-based IPS proposals already described. The table presents only those works that reported their achieved accuracy using either the median ($Q_{2}$ in the table) or the mean (μ in the table). Also, the selected works reported the dimension of the environment, how the beacons were deployed and configured. We consider that a more comprehensive survey for BLE is required. Apart from the comparison of BLE to other technologies, such as in the case of [111], the survey should pay attention to proposals that provide, at least, the characteristics compiled in Table 2, in order to make comparisons easier for the reader.

The accuracy reported by the selected works is 1 m to 3 m, which is below the accuracies stated in surveys such as Basiri et al. [11]. It is reasonable for a survey to choose conservative numbers when stating accuracy ranges, as the values reported by the IPS proposals are commonly for specific experimental settings. Five out of seven IPS proposal referenced in Table 2 correspond to small environments. For the same number of deployed beacons, the accuracy obtained in a small environment is likely to be smaller than the one obtained in a medium or large environment. Also, the selected proposals tend to have used uniform beacon deployment, i.e., beacons were placed at regular distances covering the environment or at its boundary edges.

### 3.9. Radio Frequency Identification (RFID) and Near Field Communication (NFC)

The main components of RFID are electronic tags that store some data—usually an ID—and readers capable of obtaining through RF the data from those tags. The tags can be passive, active or semi-passive. Passive tags use energy from the reader’s signal to transmit their data. Active tags have batteries and broadcast their data periodically. Semi-passive tags broadcast their data only upon the detection of a reader’s signal [46].

As readers are usually larger and more expensive than tags, one common setting in RFID-based IPS is to deploy a large number of tags across the environment and have the readers carried on, or being attached to the positioning subjects [13,43]. The setting where readers are fixed, and tags are carried the positioning subjects is more convenient for supporting large numbers of positioning subjects [46]. The detection range of the tags depends on whether they are active or passive. For passive tags, the detection range also depends on the reader’s signal power. The reported accuracy in RFID varies significantly, and it depends on how dense the deployment of tags or readers had been, with some works reporting accuracies of a few centimeters [46]. Most of the RFID-based IPS are based on simple proximity or RSS-based lateration [43,46].

NFC-based IPS have been considered to be a variant of RFID-based IPS [46]. NFC allows two devices, usually smartphones, to communicate while in touch or close proximity. A few recent IPS proposals [13] have harnessed the NFC capability of many modern smartphones in combination with the deployment of NFC tags across an environment. However, such systems have the inconvenience of requiring the active participation of the positioning subject, who is required to approach the smartphone to the tag. Thus, such systems are unable to provide continuous position estimates [13,46].

The review presented in Shen et al. [43] is very brief (less than 20 references), even considering its date of publication and the fact that the number of RFID or NFC proposals is small when compared to, e.g., WiFi. In recent years, novel RFID-based IPS proposals, such as Sakpere et al. [114], Xu et al. [115], Seco and Jiménez [116], Xu et al. [117], Yao and Hsia [118] to cite a few, have been published. Thus, a new comprehensive survey is suggested. That survey should not only review recent academic work about RFID-based IPS, but also look for its usage in commercial IPS solutions. RFID-based IPS proposal should still be appear in the following years, given the small cost of RFID tags and its suitability for assets tracking.

### 3.10. Other Technologies and Particular Cases

Cellular networks, in the form of GSM, LTE, 5G or other, are frequently mentioned but rarely addressed in detail in the selected surveys. Cellular networks have been used for positioning [12,20,45,59] applying proximity, RSS fingerprinting, and observed TDOA lateration, but not necessarily for indoor scenarios. Indeed, with reported accuracies that are commonly above 50 m, there are no IPS that are based solely on cellular networks signals to the best of this work’s knowledge. Thus, this technology is used as a signal of opportunity in conjunction with others such as WiFi, BLE and, FM.

Basiri et al. [11] refers to other technologies such as high sensitivity GNSS receivers, pseudolites, and tactile sensors. It explains that the first two are expensive and the third one is harder to manage in crowded scenarios. The tactile sensors placed on the floor may have real applications. Their sensing capability is provided by a relative simply technologies such as piezoelectric sensors, buttons or capacitive touch surfaces that can provide high accuracy in an unobtrusive way to the user. Xiao et al. [45] comments on the usage of FM radio signals for positioning, being mainly applied using fingerprinting. Even though Xiao et al. acknowledges advantages of FM-based positioning such as low power consumption in smartphones and robustness to obstacles, it cast doubts on FM usage for IPS devoted to smartphones because of the lack of RSS readings availability.

Wireless Sensor Networks (WSNs) do not constitute a technology upon which IPS are supported. However, they are commonly mentioned in the selected surveys as they may be applied to indoor scenarios and network node position determination is a relevant problem for them. A WSN is a collection of nodes able to communicate among them—or at least to the nearest neighbors—and perform some sensing task [58]. If the positions of some nodes are unknown, the communication technology or some extra ranging capability is usually used for determining the nodes’ positions [60]. If a ranging capability is used, then the TOA, TDOA, AOA and RSS techniques are used with the angulation and lateration methods, among others. Ranging may be energy prohibitive for some WNS, which thus mostly rely on interpreting the connectivity information between the nodes, i.e., using hop count [20].

Much like WSN, a highly heterogeneous network of devices organized under the concept of the Internet of Things (IoT) uses the connectivity or ranging capabilities of their nodes for position determination [15]. However, in IoT, the node’s connectivity is generally less restrictive than in WSN. Thus, IoT is expected to boost the device-free positioning to enable applications such as smart environments [22,59,68].

ZigBee is a standard for WPAN focused on providing short-range, low power and low data rates communication. ZigBee is prone to interference from signals operating at the same frequency. It has been used for device-based IPS using the RSS, TDoA, ToA and AoA techniques [22,45,46]. It has also been used for device-free positioning by analyzing the signal fading induced by human movement [45].

Among the technologies with less academic publications on IPS proposals, 5G is the one that draws the largest expectation. For some, it may be the definitive answer to most IPS applications. However, it is still at an early stage of deployment. Despite it already exits surveys on 5G-based positioning [119], IPS based on non-experimental 5G networks are not a reality yet. Thus, a survey in the topic will have higher value if it is created after the deployments of 5G networks and their support in modern smartphones notably increase.

### 3.11. Accuracy Summary of Technologies

Table 3 presents the typical accuracies reported for the technologies most commonly used for IPS. The accuracy values were inferred from values reported in the selected surveys, either from those whose main topic was the specific technology (“Main S.”) or those that only briefly addressed the technology (“Sec. S.”). The table also includes notes on the surveys, stating whether they provided accuracy summaries for comparisons or any particularity of their summaries.

Thought accuracy is a key factor in the election of an IPS, it is not the only factor. Usually, an important factor is whether the IPS will apply to smartphones or not. The ones providing the highest accuracies are commonly not applicable to smartphones. The technologies that can provide the best accuracies are Light, Sound, UWB, Artificial Magnetic Fields, and Computer Vision. The remarkably high accuracy of light technologies is mainly achievable under specific techniques (TDOA), equipment (photodiode array) and conditions (LOS). The accuracy of (Ultra)Sound, UWB and Artificial Magnetic Fields technologies is also high. However, they require techniques (TOA, TDOA, and AoA) that are not supported by off-the-shelf smartphones. Almost every modern smartphone has an integrated camera, thus enabling the application of computer vision. However, using the camera for position determination requires the user to focus its attention on the position task and orient the smartphone accordingly. Natural Magnetic Fields, WiFi, BLE, and PDR are technologies commonly applied to IPS that support smartphone applications. The accuracy of PDR drops as the distance from the last fix (known position) increases, thus it is most commonly used in combination Magnetic Fields, WiFi, or BLE. The Magnetic Fields-based and WiFi-based IPS for smartphone applications commonly require the collection of a fingerprint database, which is a very time-consuming process. However, they are popular choices because they do not require the deployment of any hardware. The BLE technology provides better accuracies than WiFi and natural magnetic fields, at the expense of a relatively cheap deployment of BLE beacons. Other technologies such as RFID, Cellular, WSN or ZigBee are less considered than BLE or WiFi, although they can achieve comparable accuracies. The position determination based on cellular network has low accuracy. WSNs are per se a specific type of application that is not commonly linked to smartphones. The RFID and ZigBee technologies require particular hardware deployments that vary regarding range, receptor requirements, and cost. Thus, they have not had the success of other technologies such as BLE or WiFi.

## 4. WiFi and BLE RSS Fingerprinting for Smartphone-Based IPS

The IPS solutions applied to smartphones normally use technologies that depend on the propagation of RF signals in indoor environments. The indoor environment, however, is not benevolent for RF signals. Multipath originates because of reflections of the signals on obstacles. The multipath appreciated outdoors at a large scale is more significant indoor, given that obstacles are abundant and close to each other. In multipath, a signal pulse reaches a receiver as several components that may have an additive or subtractive effect on the signal power [8]. The effect of multipath depends, at least in part, on the bandwidth of the signal pulse. As reviewed in Section 3, UWB is less affected than WiFi, which in turn in less affected than BLE. Additionally, all measurements are affected by the random noise that results from, e.g., thermal and circuit noise. Also, the collisions that result from using a shared medium are common [27]. Furthermore, the human body significantly attenuates the signals from the 2.4 GHz band [151], which leads to obtaining measurements that notably differ depending on the receiver device orientation when it is held by a person.

The above challenges, in conjunction with the common difficulty of knowing the actual position of RF emitters, hinders the obtainment of proper accuracies for lateration methods. Thus, RF RSS fingerprinting has become widely popular among the solutions for smartphone-based IPS, mainly for those using the WiFi or BLE. Fingerprinting is applied using either deterministic, probabilistic or machine learning approaches [19,48].

The deterministic approach commonly refers to the k-Nearest Neighbors (kNN) method or a variant of it. In kNN, the new fingerprint used for position estimation is compared for similarity against fingerprint values previously stored in a database. The positions associated with the k most similar fingerprints are used to infer a position for new fingerprint. Apart from the k value and the way that the k positions are combined, the distance metric used for determining similarity among fingerprints is an important aspect to consider in kNN [152].

In probabilistic approaches, the stored fingerprint values are used to compute the probability distribution of the signal of each emitter at each point. Those distributions are used later to select the most likely positions for the new fingerprint using Bayesian theory based on signal strength. Given that the Gaussianity of RSS behavior is commonly acknowledged, the computation of the probability distribution normally determines the μ and σ parameters of a Gaussian distribution. However, some studies state that Gaussianity assumption does not necessarily hold [19]. Probabilistic approaches include Bayesian networks, expectation-maximization, Kullback–Leibler divergence, Gaussian processes, and conditional random fields [17].

The machine learning approaches harness the development of methods and tools created in recent years for the field of Artificial Intelligence. The radio map is used to train Support Vector Machines (SVM), Artificial Neural Network (ANN), among others [56], normally in a supervised fashion. Then, the trained models are commonly used for regression on 2D positions, and classification for floor or building estimates.

It is acknowledged that IPS based solely on WiFi fingerprinting can offer 2 m to 3 m of mean accuracy, although most common figures show 6 m to 7 m. BLE can provide better accuracies than WiFi, in the order of 1 m to 2 m, but larger values are also possible [105]. The variations in the previous numbers not only relate to specifics of the approach applied, but also to the characteristics of the environment and the collected training fingerprints. Indoor environments are not equally detrimental for RF fingerprinting, varying significantly in the number and materials of the obstacles, the influence of electronic equipment and the dynamics of the people moving around. Also, the number and disposition of the emitters are highly relevant [56,153]. An increase in the number of emitters (WiFi AP or BLE beacons) found in a scenario can improve IPS accuracy [105]. Furthermore, relying only on distant emitters leads to little differentiability among fingerprints of distinct reference points, thus deteriorating the accuracy.

The number and distribution of reference points can also influence the obtained accuracy. On one hand, large mean errors are expected for sparse collection over large environments [154], assuming that no densification strategy is used. On the other hand, the mean error tends to be small in small environments that are densely surveyed [155]. Additionally, the devices that measure the signals—the chipsets in smartphones—have different levels of sensibility [17] and identify the signal strength in distinct ways [56]. Thus, the strength reported for the same signal may have distinct values for distinct devices. Furthermore, one device may measure and report the RSS of a signal while another device may not report any value for the same signal because the signal strength is beyond its sensibility threshold. The RSS values measured at a given point are also affected by the device orientation [56], which is a result of the type and disposition of the antennas in the device and the partial absorption of the WiFi and BLE signals by the human body.

The previous difficulties have long been known for studies on WiFi, and later BLE, fingerprinting IPS solutions. To cope with them, the most straightforward strategy is to collect for the target environment a large database of fingerprints that covers as much as possible the given scenario, that has as many as possible reference points and that collects several fingerprints at several directions using distinct devices. However, such collection is very time-consuming. Apart from being cumbersome, it is not affordable sometimes. Therefore, one of the main challenges for (particularly WiFi) fingerprinting positioning has been the effort reduction for collecting the training fingerprints. The proposed solutions for alleviating the collection efforts mainly include:

- Do the collection using crowdsourcing.
- Apply a propagation model to estimate the expected RSS values.
- Perform a small site survey and apply regression techniques to enrich the radio map.

The crowdsourcing approach harness the potential of the explicit or implicit participatory actions of users [19]. In the explicit modality, the users manually tag the positions of the recorded measurements. The users’ participation can occur during the offline stage or the online stage. During the offline stage, the participants supply the position tags required to have an operational positioning service [19]. During the online stage, the participants only supply position tags when the system is unable to provide an estimate [25]. In the implicit modality, the user is not requested to provide position labels. Instead, the labeling is performed by the positioning system whenever is possible using occasional fixes provided by, e.g., the GNNS receiver. Also, the IPS can infer fixes using heuristics or learning methods based on the existing radio map and inertial measurements [19,25].

The approach of creating the radio map by applying a propagation model is attractive and has been proposed [56]. However, this approach requires either previous information on the positions and broadcasting parameters of the emitters, or to estimate that information using a few samples. The radio map densification using regression has been effectively used in several studies using regressors such as linear regression, nonlinear Gaussian Process, Gaussian Kernel Learning, and augmented path-loss model, Support Vector Regression, and Random Forest [19,156,157].

Apart from the radio map creation, another acknowledged challenge in WiFi or BLE fingerprinting-based IPS is the energy consumption reduction [6]. WiFi scans are very energy demanding. BLE scans demand less energy than WiFi scans. Furthermore, the deterministic approaches must deal with large databases for large scenarios, which implies performing many comparisons of vectors of potentially large dimensionality. Pérez-Navarro et al. [6] and He and Chan [17] mention several approaches to deal with the former issue. Also, Khalajmehrabadi et al. [19] mentions approaches to deal with large fingerprint databases. Those approaches are mainly based on techniques such as clustering by Binary AP Coverage, K-means clustering, Affinity propagation, and spectral clustering.

Other acknowledged challenges are to detect and adapt to changes in the infrastructure that affect the positioning [17], which is also related to radio map construction; the detection of outliers [19]; the device diversity issue, i.e., the adaptation to heterogeneous devices [19]; the proper evaluation of IPS proposals [6,48]; and the accuracy improvement. Solutions based solely on WiFi or BLE have known accuracy limitations [6]. Accuracy improvement is the challenge most often addressed. It is not commonly addressed for approaches based solely on WiFi or BLE, but for solutions that combine several technologies and techniques. For example, WiFi or BLE is normally combined with PDR, and the position estimates are corrected using filters such as Kalman or particles filter [56] and map-matching [6,20].

Section 3 already mentioned survey works specifically devoted WiFi fingerprinting [17,19,56]. Other surveys such as Pérez-Navarro et al. [6] addressed fingerprinting across several technologies. A trait common to all of them is that, when addressing the reduction of the effort for radio map creation, they devoted more content to crowdsourcing-based solutions than to radio map enrichment. Crowdsourcing has been called to be the most promising solution for radio map creation, and thus several surveys have addressed this topic.

Pei et al. [42] focused on crowdsensing (implicit user participation). It reviewed the most relevant crowdsensing works for indoor positioning using opportunistic signals by the time of the study. The study is a recommended reading because it follows a clear order on explaining signals of opportunity and how to infer position tags for collected data (e.g., using dead reckoning and indoor maps) and its relation to fingerprinting for indoor positioning. The survey presented in Zhou et al. [63] gives an introduction to indoor positioning and devote sections to (mainly WiFi) crowdsourcing, automatic construction of floor plans, self-deployable indoor positioning and navigation systems and their associated challenges. Lashkari et al. [68] provides a survey on crowdsourcing that, despite a slight IoT flavor, is comprehensive and addresses solutions not conceived originally for IoT. The survey differences crowdsourcing (explicit user intervention) from crowdsensing and it explains detailed insights into each considered solution.

### Radio Map Enrichment Approaches

Currently, no survey has devoted at least a large part of its content to radio map enrichment. Thus, we provide a brief review of selected academic works. The radio map enrichment methods can be classified into four large groups: methods based on sparse recovery, interpolation or extrapolation methods, propagation models, and regression methods. Only the first group has a small number of proposals. Examples of the first group are Gu et al. [158] and Khalajmehrabadi et al. [159]. Gu et al. [158] applied compressive sensing using Singular Value Decomposition (SVD) formulated as an optimization problem to reduce the sparsity of the output matrix. Also, the authors applied kNN to infer some extra measurements in the matrix. Khalajmehrabadi et al. [159] performed radio map interpolation using sparse recovery, which employed a Fourier transform and minimization using sparse group Lasso.

Remarkable examples of the second group are the evaluation of alternatives performed in Ezpeleta et al. [160] and Talvitie et al. [161]. Ezpeleta et al. [160] considered interpolation of RSS signals, using ZigBee. As interpolators, the authors considered radial basis functions: Euclidean distance linear, multiquadratic, thin plate spline and polyharmonic spline, being thin plate the best performing one. Xie et al. [162] also tested RBF interpolators, considering several shape parameters for a multi-quadric function on BLE samples. Talvitie et al. [161] tested, for each floor and AP, a combination of Linear Interpolation with extrapolation methods based on the minimum detected value, the mean detected value, and on the signal gradient on triangulation edges. Also, the authors applied the Nearest Neighbor and Inverse Distance Weighting (IWD) methods, which can be used directly for interpolation and extrapolation. The IWD method consistently provided the best recovery results across the analyses. Another example in this group is Bong and Kim [163], which used an interpolation for WiFi signals based on discontinuity preserving smoothing. According to the authors, the method preserves discontinuity over walls in accordance with the signal gradients. Other examples are Zhang and Cai [164], where a multivariate polynomial interpolation was tested using simulations; Racko et al. [165], which tested Linear and Delaunay triangulation-based interpolations; Chai and Yang [166], which performed linear weighted interpolation of RSS distributions, for probabilistic fingerprinting, instead of interpolating the RSS values; and Moghtadaiee et al. [167], which placed samples and estimation points into rings according to the distance from the AP, and used the mean of the samples in a ring as the value for the estimate points inside that ring.

The third group (propagation models) includes many proposals. One of the models commonly used is the log-distance path-loss model Seybold [168]. Ali et al. [169] applied the path-loss model considering a fixed wall attenuation factor and distinct values of the path-loss coefficient differed for LOS and NLOS situations. Narzullaev and Park [170] modified the log-distance path-loss model to consider the power and distance associated with the sample point closest to the AP instead of the traditional power of AP at close distance (usually 1 m). He et al. [171] applied the log-distance propagation model, assuming knowledge of the AP positions, to estimate RSS at points lying in the lines defined by collection points and APs. Moghtadaiee et al. [167] proposed to fit the path-loss model independently in each zone delimited by architectural divisions, assuming knowledge of the APs position, which performed better than the other tested interpolation methods. Li et al. [172] fitted a log-distance path-loss model for each target position, giving distinct weights to samples in the fitting process according to their distances to the target position.

The ray tracing model and the radiosity model are another two techniques used for modeling RF propagation [173,174] that have been used for RSS database creation. Ayadi et al. [175] tested the ray tracing and radiosity models along with the log-distance path-loss model and the Cheung model [176]. The ray tracing and radiosity models performed significantly better than the other two regarding mean and the standard deviation of recovery error. The work of Belmonte-Fernández et al. [177] also tested the radiosity model for WiFi RSS radio map creation in a floor of an office building where the authors deployed four WiFi APs.

The group of regressions also has many proposals. In particular, the Gaussian Process Regression (GPR) has been widely used. Faragher and Harle [87] used GPR for BLE radio map enrichment. Sun et al. [178] evaluated GPR using six distinct kernel functions and some combinations of them. Richter and Toledano-Ayala [179] suggested the usage of GPR with constant mean function and Matérn class covariance function, instead of the commonly used zero mean squared exponential kernel. Atia et al. [180] proposed to first fit a log-distance path-loss model and then fit GPR on the residuals produced by the log-distance model. Zou et al. [181] applied a similar approach than Atia et al. [180], but fitting a bidimensional second-degree polynomial function instead of the log-distance model. Ai et al. [182] and [183] applied GPR over samples from in route-based (walking) collection for BLE and WiFi samples, respectively. Li et al. [184], Liu et al. [185], Jan et al. [186], Kram et al. [187] applied Kriging, which can be seen as a variant of GPR. Other works using regressions are Du et al. [188], which used Geography Weighted Regression (GWR) for WiFi samples, and Hernández et al. [157], which used Support Vector Regression (SVR).

Table 4 present examples of the accuracies reported by four proposals. The table contain one example per group because there is a wide variety in the experiment settings and reported metrics among proposals, which make comparison difficult. For example, Ayadi et al. [175] tested several propagation models, but considered RSS differences instead of absolute RSS difference, and thus the reported mean values are close to zero. Even within the selected work, we could not find all target information. The position accuracy using real samples instead of estimated intensities could not be inferred for Narzullaev and Park [170] and Sun et al. [178]. For them, it is difficult to tell how much the reduction in the number of collected samples affected the positioning accuracy. The solutions shown in Table 4 reported tests in a building, and the tag of “Medium” or “Large” indicate that the test included 1 or 4 floors, respectively. Only Talvitie et al. [161] performed experiments in a large environment with a large number of detected APs. In general, recovery accuracy values higher than 5 dBm seem common when using 20% of for fitting the models or functions and 80% for test estimates. Apart from the sparse recovery group, the recovery accuracy seems similar among the groups. The purpose of Table 4 is not only to show some information about radio map enrichment proposal, but also to highlight which are the most relevant characteristics of such proposals. One of the traits that may have not given enough importance is the selection of training and test samples to validate a proposal. While [159,178] selected random points for training and testing, [170] did a manual selection and [161] assured that there were large areas with no training points create challenging conditions for interpolation and extrapolations methods. Also, the best performing method regarding recovery accuracy is not necessarily the best performing regarding positioning accuracy [161].

## 5. Discussion on IPS Current State

Researchers working in the field of indoor positioning share the notion that one of the traits of the current status of IPS is that there is no clear prevalent technology or method for IPS. The variety of environments and applications makes it difficult to find a general solution applicable to most situations. Some surveys include evaluation metrics to compare the reviewed works and inform the reader about the characteristic of the most notable works selected by the survey’s authors. Accuracy, cost, and scalability are among the most common metrics found in the surveys, as mentioned in Section 3.

Another trait of the current status of IPS is the large number of solutions that have been proposed along the years, while only a few have had large academic significance. To deepens in the exploration of this trait, this paper compiled and linked the publications referenced by the surveys introduced in Section 3. The linkage is based on DOIs [189]. The idea is to construct an undirected graph that links the surveys and the publications referenced by them, using the DOI associated with each of them as link information. We acknowledge that this approach does not include every single academic paper. There are old papers, papers published in low profile conferences, technical reports, patents, books and online resources that do not have DOIs assigned to them. During the curation, there were publications [190,191,192,193,194,195,196,197,198,199] with no DOI that were cited up to 11 times in the selected surveys. Most of the publications with no DOI were cited two or three times in the surveys. The DOI approach was used given that it is relatively simple and allows for automatic analysis. Furthermore, most recent academic papers published in known resources are given a DOI.

**Figure 3:**
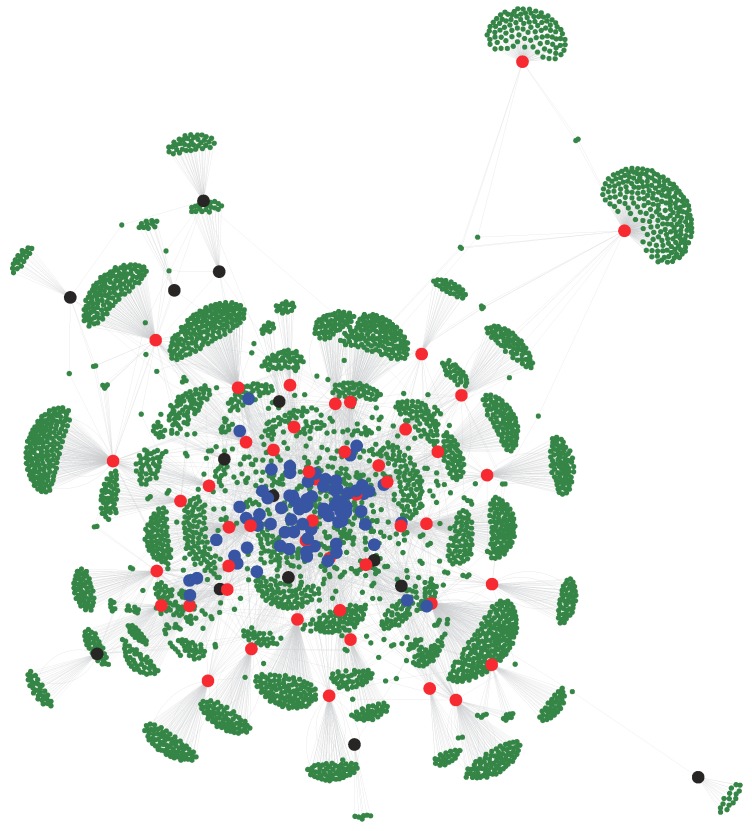
Graph of surveys and their referenced works. The selected surveys are identified by red (journal-published) and black (conference proceedings-published) dots. Their referenced publications are represented by blue (more than 5 citations) and dark green (5 or less citations) dots.

The curation was performed using automatic and manual means. The automatic means included web scraping for the reference extraction and DOI discovery using the Crossref’s Link References matching tool [200]. After the application of automatic means, a manual revision was performed to correct inconsistencies produced both by the web scraping and by the DOI discovery processes. The processing of the 62 selected surveys resulted in 3943 unique works, including surveys distinct from the 62 original ones. Figure 3 shows the resulting graph applied to all surveys, visualized using a force-directed approach [201]. Some surveys stand apart from others and lie in the outer parts of the image. Those divergent surveys are those with narrow focuses such as SLAM [16], visual odometry [74], RFID [43], multidimensional scaling techniques [60], and those published in conference proceedings [23,26,32,58]. It is of relevance to remark that some conferences apply a strict page limit, which in most of cases make the author to shorten the list of references to the most relevant ones. The surveys lying close to the center of the graph are those that reference works that are commonly referenced by other works. Those surveys mainly address WiFi-based solutions [17,34], solutions that avoid or reduce the site survey efforts [25,66], inertial sensors solutions [31], WLAN-based solutions [19], solutions specific to pedestrians or smartphones [47,48] and general surveys [14,45,46].

Figure 3 highlights the works that were cited more than 5 times by the selected surveys. The number of citations of a work is determined by the degree of the node that represents it in a graph, i.e., the number of its incident edges. The number of highlighted works is 62. It is a rather low number compared to the total number of referenced works, even without taking into account the selected surveys. Indeed, the mean number of citations of a work without considering the selected surveys is 1.46. Such low number is significant not only because it hints at that most publications are cited only once, but also because the considered works also included surveys—which were published before 2015 and therefore were not among the selected surveys for this study.

Table 5 presents the number of citations of non-survey works, i.e., those that were not among the selected surveys, they do not include in their title the words “survey” or “review”, and were not known to be a survey. Most of the referenced publications are only cited once in the selected surveys, while some of them are cited twice. The number of works that are cited more than five times is very small. The distribution in the number of citations might suggests that (1) the surveys tend to be very specific and barely intersect in content, (2) the proposed solutions quickly get obsolete or (3) it is hard for surveys in IPS to assess the relevance of the proposed solutions. The weight of the first conjecture seems to be low, given that although some of the surveys are very focused, such as Saeed et al. [21], most of them deal with several techniques, which should result in a large number of intersecting works. The weight of the second conjecture seems more significant than the one from the first conjecture. Although the obtained accuracies and the acknowledged challenges [6] have remained similar for several years [25], authors may try to incorporate the latest proposals to add value to their surveys, regardless of the actual importance of the proposals. The third conjecture is supported by works on IPS that plead for better evaluation procedures and reproducible research [10]. With so many proposed solutions, the value of a work is not always correctly assessed without a proper evaluation that includes tests on publicly available data featuring well-known characteristics. The lack of usage of a common objective evaluation framework may lead to unworthy citations or simply the dispersion appreciated in Table 5 and Figure 3.

The works that had the largest number of citations were classics of the IPS literature: RADAR [202], Horus [203], LANDMARK [144] and Zee [204]. RADAR (28 citations, year 2000) is the best known of the first IPS solutions that applied WiFi-based fingerprinting, so it is commonly cited when a proposal deals with RSS fingerprinting, mainly for WiFi or BLE. Horus (17 citations, year 2005) is also one of the best known first applications of WiFi fingerprinting. The difference between RADAR and Horus is that the former applied a deterministic approach, while the latter used a probabilistic one. LANDMARK (15 citations, year 2003) is an early IPS based on RFID. Zee (15 citations, year 2012) is an early solution to the radio construction effort, which is one of the most acknowledge challenges of fingerprinting. It successfully combined PDR with map-matching to make the WiFi radiomap grow with self-tagged samples. Other works that have more than 10 citations are related to IPS based on UWB [205]; PDR and map-matching [206]; a combination of light, sound, WiFi and inertial sensor inputs [207]; WiFi without site survey [208,209]; a combination of WiFi, magnetic, PDR and inertial sensor inputs [210]; visible light [211]; and RF and ultrasound [78]. Apart from the value of their respective contributions, the previous works may be highly cited because they were early proposals of IPS using solutions that were novel at their times.

**Figure 4:**
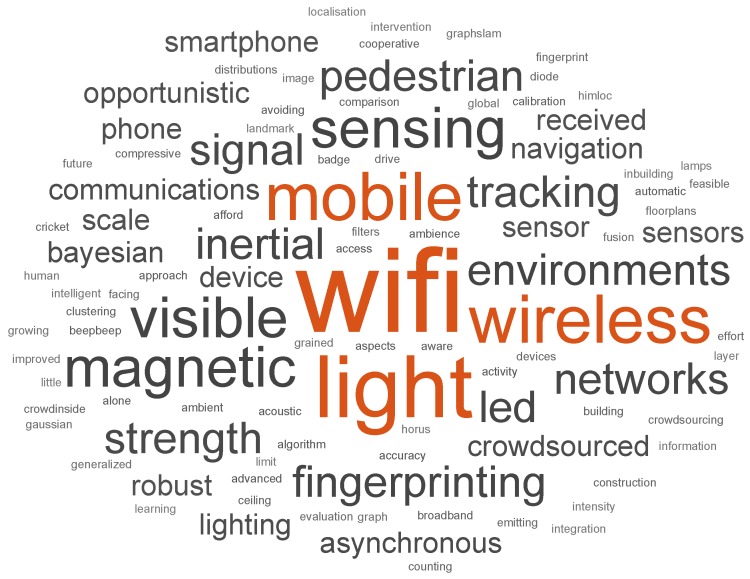
Word cloud of the works most cited (more than five citations) by the selected surveys.

To analyze the focus of the selected surveys regarding technology, they were assigned a category. The selected categories and the number of surveys that belong to each category are presented in Table 6. Despite the fact that the data from the table may suggest that light-based IPS are as popular as WiFi-based IPS, many of the surveys that were classified in the category “Several” do not deal with light-based IPS, either because they are restricted to network-based technologies or technologies that are fully supported by smartphones. Furthermore, the category “Several” include surveys that are not devoted WiFi but focus on topics that are very relevant to WiFi-based IPS, such as crowdsourcing, fingerprinting, radiomap construction, and IPS for smartphones. To further explore the focus of the current IPS research, this study extracted the 55 non-survey works most cited (more than 5 citations) in the selected surveys. We excluded surveys among the 55 works because our focus was on IPS proposals. Furthermore, surveys tend to have many citations because they are used as references on general descriptions on a subject and they are acknowledged by later, related surveys. For example, the work of Hui et al. [212], published in 2007, have 33 citations in the selected surveys. In Google Scholar [213], it had over 4000 citations by the time this paper was written. Figure 4 shows the result of taking the titles of the extracted works [75,77,78,80,82,121,133,136,139,144,202,203,204,205,206,207,208,209,210,211,214,215,216,217,218,219,220,221,222,223,224,225,226,227,228,229,230,231,232,233,234,235,236,237,238,239,240,241,242,243,244,245,246,247,248] and creating a word cloud that highlights the occurrence frequency of each word in the titles of the extracted works. Punctuation signs as well as words non-relevant for comparison such as ‘indoor’, ‘system’ or ‘and’ were removed.

The image presented in Figure 4 gives further support to the notion that WiFi is the technology most prevalent among IPS proposals. WiFi seems to be followed by light and wireless technologies—in which WiFi is also included. The 55 non-survey most-cited works were published before 2017, and almost half of them belong to the period 2011–2013. The trend in their publication years was expected given they are mostly pioneers in the indoor positioning field which have maintained their research value. It is difficult to forecast how long will they remain valuable. WiFi, BLE, and light seem to be the front-runners until a new technology—at least cheaper than UWB—becomes widely available for its use with smartphones. The chosen reviews that focused on light are from the years 2016–2019, which highlights the increasing importance that is given to this technology. Regarding WiFi, as long as smartphones keep being able to perform WiFi scanning at reasonable rates to provide near real-time positioning, research on WiFi-based IPS will persist given it is a technology that requires no infrastructure apart from the pervasive AP already in place. The new Android version (9.0) [89] restricted the number of WiFi scans to a maximum of 4 scans every 2 min. WiFi scanning was already restricted for iOS phones [249]. Thus, the restriction for Android devices should decrease the importance given to WiFi in some IPS solutions. The new scan rate is too low for pedestrian navigation solutions based solely or mainly on WiFi. However, some applications such as in-home monitoring and presence detection for data analysis only require coarse non-real-time position estimates and they should have little affectation from the new scan restrictions. BLE, which provides better estimates than WiFi, will also remain on the choices for IPS. Indeed, it is popular among IPS provider companies.

**Figure 5:**
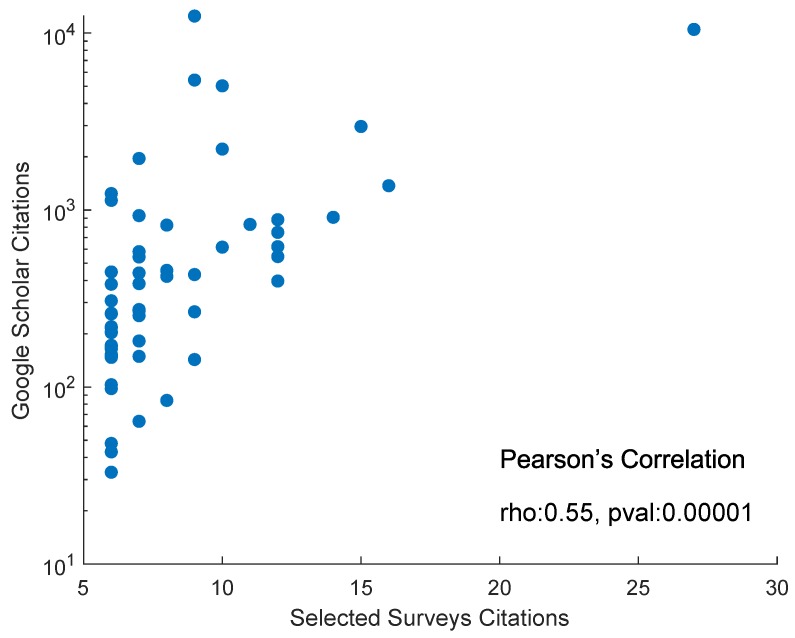
Number of citations in selected surveys vs citation from Google Scholar for the 55 non-survey most-cited works. A logarithmic transformation was applied to the y-axis to reduce the represented distance among data points.

The final batch of analyses from this section included the number of citations according to Google Scholar of the 55 non-survey most-cited works. Figure 5 contrast the number of citations according to the selected surveys and the number of citations according to Google Scholar. Also, a Pearson correlation test was applied to the two citation measures. The correlation results indicate a moderate, positive and statistically significant correlation between the two citation measures. However, some works with a small number of citations in the surveys may also have many citations in Google Scholar. An example is the work of Arulampalam et al. [214], which provided a tutorial on particle filters. That work has only 9 citations in the surveys but over 10,000 citations in Google Scholar. Although it is applicable to IPS, it does not focus on IPS and it is relevant for a broad spectrum of topics related to positioning and navigation. The work that introduced the RADAR IPS [202] has fewer citations in Google Scholar that the work of Arulampalam et al. [214], despite the fact of having 28 citations in the selected surveys.

The trend visible in the scatter plot from Figure 5 shows that works with many citations in the surveys tend to have a large number of citations in Google Scholar. An additional search for works in indoor positioning was performed in Google Scholar to find works with many citations that were not referenced in the selected surveys. The search resulted in a few works [250,251,252,253,254,255,256,257] published before 2011 with around 200 to 400 citations according to Google Scholar. Numbers of citations between 200 and 400 are not highly significant given that the median value of Google Scholar citations for the 55 works is almost 400. Thus, the notion that a work that has many citations from surveys is likely to have a large number of citations in Google Scholar is further supported.

**Figure 6:**
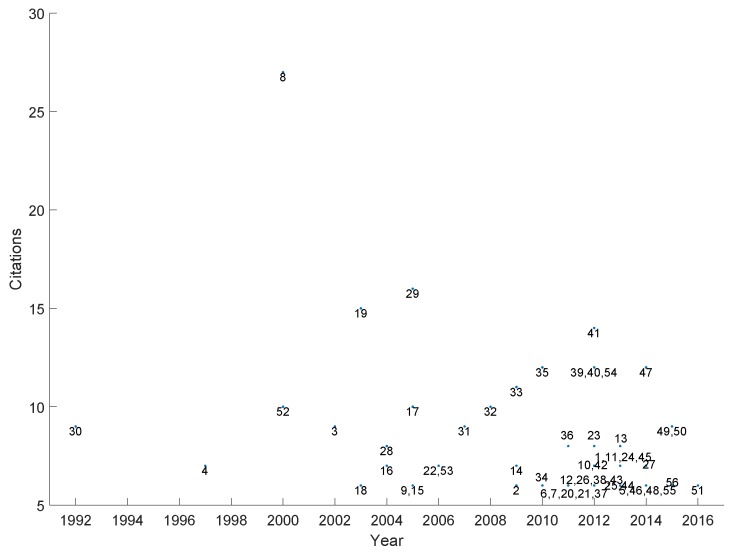
Citations in the selected surveys of the 55 extracted works. The x-axis represents the year of publication of the work.

**Figure 7:**
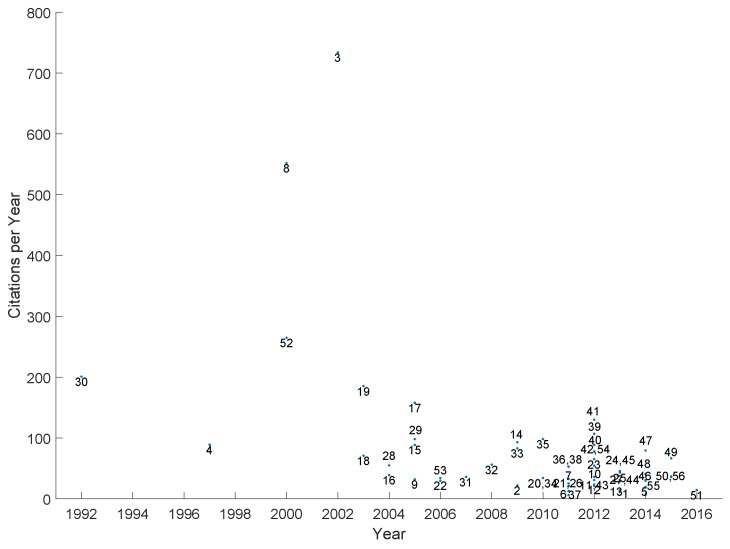
Mean number of citations per year in Google Scholar of the 55 extracted works. The x-axis represents the year of publication of the work.

Figure 6 explores the relation of the number of citations according to the selected surveys with the year of publication of the extracted works. Figure 6 shows that, regarding the selected surveys, the number of citations of a work does not depend on the number of years since its publication. The works corresponding to data points 35, 39, 40, 41 and 47 [204,208,209,210,211], published in or after 2010, have more citations than all other works apart from those corresponding to the data points 8, 19 and 29 [144,202,203]. Further analyses regarding the number of citations from Google Scholar showed a steady decrease in the number of citations along the years. Such decrease creates difficulties to assess the importance of a published work. Thus, Figure 7 presents the mean number of citations per year in Google Scholar. Figure 6 and Figure 7 used the same numbers for identifying works. Some works that Figure 6 showed to be relevant, such as those from data points 8, 19, 39, and 40, are still noticeable in Figure 7. However, the mean number of citations per year seems to decrease along the years, thus suggesting that the mean number of citations per year for a published work may indeed increase every year.

Finally, 62 new IPS works from 2018 with 5 or more citations [91,116,117,118,177,178,258,259,260,261,262,263,264,265,266,267,268,269,270,271,272,273,274,275,276,277,278,279,280,281,282,283,284,285,286,287,288,289,290,291,292,293,294,295,296,297,298,299,300,301,302,303,304,305,306,307,308,309,310,311,312] and 46 new IPS works from 2019 with 1 or more citations [105,313,314,315,316,317,318,319,320,321,322,323,324,325,326,327,328,329,330,331,332,333,334,335,336,337,338,339,340,341,342,343,344,345,346,347,348,349,350,351,352,353,354,355,356] were selected using Google Scholar. The selected works from 2018 were mainly published in periodic journals, although some works from conferences proceedings were also included given their high number of citations. The works were found through Google Scholar queries, using the search terms “indoor positioning OR indoor localization”. Table 7 presents the aggregated numbers of works by their main applied technology—the one that provided position fixes, if any—and by the two selected years. The table does not account for works that did not consider a specific main technology or the technology was not typically addressed in indoor positioning works (e.g., pseudolites). We decided to perform a general IPS query (instead of one per each technology) and to apply a filter on the number of citations to reduce the number of references added to this manuscript. The numbers presented in Table 7 support the previous notions that WiFi-based and light-based solutions dominate the published IPS academic solutions. The number of WiFi-based works is only slightly higher than the number of light-based works. BLE works also have a significant presence in IPS publications. For most technologies, the number of works from 2018 is higher than those from 2019. Despite the filter for 2018 is more restrictive than the one for 2019, the works for 2018 have been available for reader (and thus for citations) during more time. For the WiFi, light and BLE technologies, the number of works for 2019 is almost the same as for 2019, which may be in part a result of the applied filters. However, for PDR and 5G (cellular), the number for 2019 is higher than for 2018. The case of 5G is notable, given that for 2019 it has more works than traditional IPS technologies such as Sound or ZigBee, indicating the raising relevance that 5G is gaining for indoor positioning.

According to analyses presented in this section, the set of IPS-related surveys seems to be a good mean to compile the works that had fundamental academic contributions to the IPS topic. However, each survey incorporates many works whose appreciated relevance is not shared among other surveys or even among the IPS literature in general. The number of citations is one of the metrics traditionally used to measure the scientific impact of a work in academia [357]. Furthermore, citations play important roles in academia such as acknowledging others’ ideas and provide the reader with useful additional information. Thus, relevant and known works in academia tend to have large numbers of citation. However, we acknowledge that not all citations are meaningful, and the number of citations is not the only tool to measure the significance of a work, especially in areas beyond the academia [358]. Also, we do not imply any kind of relation between the number of citations and the quality of the development presented by a work.

## 6. Conclusions

This paper presented an analysis of indoor positioning systems based on previous surveys. In contrast to traditional surveys, where the different indoor positioning technologies and state-of-the-art works are analyzed, this work analyzes the current status of indoor positioning based on the works cited and referenced in previously published surveys.

The paper addressed the main technologies that currently support indoor positioning systems, briefly stating the techniques and methods applied in each of them. The description of each technology is supported by references to other surveys, thus enabling the reader to use this paper as a collection that provides links to more specialized surveys. The paper also compiled the most-recognized challenges in indoor positioning and deepens into positioning based on WiFi and BLE fingerprinting, which is the most popular method for indoor positioning.

The meta-review shows that most of the cited papers within a review are not disruptive. A total of 3943 works (with DOI number) were cited in the context of 62 reviews, but most of them (more than 3200) were cited once within the selected surveys. On the other hand, only 55 works were cited more than 5 times in the selected surveys, being considered well-known or disruptive works. In some cases, the surveys cite recent papers that do not attract the indoor positioning experts.

Finally, the meta-review also shows that a few papers published in early 2000s were disruptive and have had a huge impact on further developments. Radar has more than 10,000 citations (around 550 average citations per year). There are only a few works with moderate impact (around 100–200 average citations per year since they were published) in the period from 2005 and 2010. Most of the relevant works in the surveys are in the period 2010–2016, which match the born of smartphones as we currently know them and the proliferation of new conferences related to the topic, such as the International Conference on Indoor Positioning and Indoor Navigation (IPIN).

## Figures and Tables

**Table 1:** Publication year distribution of the selected surveys.

Year	2015	2016	2017	2018	2019
Number	12	16	13	13	7

**Table 2:** Selected BLE IPS proposals.

REF	Method	Accuracy	Environment	Beacons	Notes
[87]	Probabilistic	$Q_{2}\approx$1 m	50×15m, several offices	19 (−12 dBm, 10 Hz)	Best of several selected deployment configurations.
[97]	Stigmergic (trail map)	$Q_{2}\approx$ 1.5 m	6×6m, 1 office	8 (−16 dBm, 3 Hz)	Deployed uniformly at environment edges.
[98]	kNN	$Q_{2}=0.77\;m$	52×43m, 1 building floor	17 (0 dBm, 10 Hz)	Combination of WiFi and BLE under one distance, using 4 WiFi APs.
[112]	Isomap and kNN	μ≈1.5 m	6×18 m	30 (−8 dBm, 2 Hz)	Uniform deployment in grid.
[100]	SVM	μ≈2 m	4×3 m	5 (−12 dBm)	Uniform deployment at the edges of the environment, LOS conditions. No adv. frequency provided.
[105]	Weighted Centroid	μ≈2 m and 2.5 m	151 m and 176 m	24 and 22 (−12 dBm, 5 Hz)	Two environments, rooms with tall obstacles. Uniform deployment.
[108]	Lateration	μ≈2 m	9×12m, a few obstacles	4 (0 dBm)	Deployment in environment corners. No adv. frequency provided.

**Table 3:** Typical accuracies of selected technologies and their related selected surveys.

Tech.	Main S.	Sec. S.	Typical Accuracy	Notes on Surveys
Light	[24,36,37,51,64,65]	[21,46]	Depends on technique and setup. From median < 1 mm [120,121] to median < 2 m [122,123].	All Light surveys provide accuracy summaries. Luo et al. [51] addresses visible light IPS and gives positioning accuracy for each type of its taxonomy. Zhuang et al. [64] divides the accuracy into 2 tables, for camera-based and photodiode.
Computer Vision	[74]	[12,13,16,46]	For odometry, from 0.25% [124] to 8.5% of path length [125]. For maker-based solutions, median < 1 m [126,127].	Aqel et al. [74] addresses odometry and mentions limitations of each method, but provides accuracy for only a few methods.
Sound	[61]	[13,45,46]	For ultrasound, median < 1 cm [61]. For audible sound, median < 10 cm [128].	Ureña et al. [61] does not provided accuracy summary.
Magnetic Fields	[50,54]	[6,12,15,46,48]	For artificial fields, median < 1 m [129,130]. For the natural field, median < 5 m [82,131].	Pasku et al. [54] and He and Shin [50] provide accuracy summaries.
PDR	[31,62,70]	[12,13,20,47,71]	0.3–1.5% of walked distance [132,133], median as low as 2 m for specific environments [134] but commonly above 5 m [135]. For SLAM, median 1 m to 10 m [136,137]	Diaz et al. [70] does not provide accuracy indications on the methods. Wu et al. [62] provides accuracy summary only for motion classification. Yang et al. [31] provides summaries for step count and heading errors, and for position accuracy of IPS mixed with Magnetic or WiFi. Laoudias et al. [20] provides accuracies for SLAM.
UWB	[30,33,52]	[21,46]	Commonly, median < 50 cm [138].	Shi and Ming [30] and Alarifi et al. [33] provide no accuracy summary, though Alarifi et al. [33] gives summary table of 39 proposals. Mazhar et al. [52] provides brief accuracy summary of a few, most relevant solutions.
WiFi	[17,19,27,34,56,67,88]	[6,15,46,48]	For fingerprinting, median < 5 m are common [19,139]. For time-based techniques, median < 2 m [140,141]. For CSI techniques median < 2 m [142,143].	Makki et al. [27] and Kandel and Yu [67] address time-based and CSI techniques, respectively, providing an accuracy summary. Konings et al. [88] addresses device-free solutions, without providing an accuracy summary. Basri and Khadimi [34], Xia et al. [56] and He and Chan [17] address fingerprinting, without providing accuracy summary of IPS based solely on WiFi. Khalajmehrabadi et al. [19] provides an accuracy summary of selected fingerprinting methods.
BLE		[11,13,15,18,46,48]	Median between 2 m to 5 m [110,111].	No survey provides accuracy measures for several BLE IPS. Davidson and Piche [48] deals with BLE in more details than the others.
RFID	[43]	[13,46]	Median < 2 m [115,144].	Shen et al. [43] provides accuracy for only two methods.
Cellular		[12,20,45,59]	Median < 50 m [145,146]	Laoudias et al. [20] presents accuracy summary for some cellular-based IPS.
WSN	[28,58]	[20,35,60]	Median < 2 m [147,148], but higher values can be found [60].	Mistry and Mistry [28] and Chowdhury et al. [35] provide no accuracy summary.
ZigBee		[22,45,46]	Median < 5 m [149,150].	No survey provides accuracy summary for several ZigBee methods.

**Table 4:** Selected examples of radio map enrichment proposals. RAC represents the recovery accuracy; OPA represents the positioning accuracy using actual measurements; and PAC represents the positioning accuracy using estimated intensities.

	Sparse Recovery	Interp. and Extrap.	Propag. model	Regression
**Method**	Group Sparsity [159]	IDW [161]	Log-distance [170]	GPR [178]
**Environment**	Medium	Large	Medium	Medium
**No. of APs**	19	422	32	6
**RP removal**	Random	Empty blocks	Manual	Random
**RAC** (dBm)	$Q_{2}\approx 11$ for 20%	$Q_{2}\approx 6-7$ for 20%	μ≈4−5 for 20%	μ≈6 for 40%
**OPA** (m)	$Q_{2}\approx 1$	μ≈5	*Not reported*	*Not reported*
**PAC** (m)	$Q_{2}\approx 2$ for 20%	μ≈12−13 for 20%	μ≈2−3 for 20%	μ≈5 for 21%

**Table 5:** Number of citations of non-survey works.

Citations	Percentage
1	80.0%
2	11.2%
3	4.1%
4	2.0%
5	1.2%
≥6	1.6%

**Table 6:** Number of selected surveys per technology.

Technology	Surveys
Light	6
WiFi	6
PDR	4
UWB	3
Magnetic	2
Sound	1
RFID	1
Vision	1
Several	38

**Table 7:** Number of selected recent IPS works by year and technology.

Year	Cit. Filter	5G	BLE	Light	PDR	RFID	Sound	UWB	Vision	WiFi	ZigBee
2019	≥1	2	5	12	4	0	1	3	2	14	1
2018	≥5	1	7	13	3	3	3	5	4	15	2

## Abbreviations

The following abbreviations are used in this manuscript:

AOA	Angle of Arrival
BLE	Bluetooth Low Energy
DOI	Digital Object Identifier
DR	Dead Reckoning
GPR	Gaussian Process Regression
IDW	Inverse Distance Weighting
IMU	Inertial Measurement Unit
IoT	Internet of Things
IPS	Indoor Positioning System
LED	Light-Emitting Diode
LOS	Line of sight
NFC	Near Field Communication
NLOS	Non-Line of sight
PDR	Pedestrian Dead Reckoning
RF	Radio Frequency
RFID	Radio Frequency Identification
RMS	Root Mean Square
RSS	Received Signal Strength
SVR	Support Vector Regression
TDOA	Time Difference of Arrival
TOA	Time of Arrival
UJI	Universitat Jaume I
UWB	Ultra-Wideband
WLAN	Wireless Local Area Network
WPAN	Wireless Personal Area Network
WSN	Wireless sensor networks
